# Early assessment of the impact of mitigation measures to control COVID-19 in 22 French metropolitan areas, October to November 2020

**DOI:** 10.2807/1560-7917.ES.2020.25.50.2001974

**Published:** 2020-12-17

**Authors:** Guillaume Spaccaferri, Sophie Larrieu, Jérôme Pouey, Clémentine Calba, Thomas Benet, Cécile Sommen, Daniel Lévy-Bruhl, Sabira Smaili, Didier Che, Laurent Filleul, Céline Caserio-Schönemann, Fatima Ait-El-Belghiti, Sylvie Haeghebaert, Jean-Claude Desenclos, Laëtitia Huiart, Anne Laporte, Patrick Rolland

**Affiliations:** 1 Santé publique France, Saint-Maurice, France; 2 These authors contributed equally to this article and share first authorship

**Keywords:** COVID-19 – Mitigation measures – Epidemic – Impact – Surveillance

## Abstract

In France, measures including curfew and lockdown were implemented to control the COVID-19 pandemic second wave in 2020. This study descriptively assesses their possible effects, also relative to their timing. A considerable decrease in incidence of COVID-19 cases and hospital admissions was observed 7 to 10 days after mitigation measures were put in place, occurring earlier in metropolitan areas which had implemented these first. This temporal coincidence suggests the measures’ positive impact, consistent with international experiences.

In spring 2020, an important means to curb the first wave of the coronavirus disease (COVID-19) pandemic in France was the implementation of a national lockdown from 17 March to 10 May [1]. Subsequently, transmission remained stable and at a low-level until the end of July. In August and September, however, a new steady rise was observed, followed by a rapid increase in severe acute respiratory syndrome coronavirus 2 (SARS-CoV-2) spread. Metropolitan areas were particularly affected. To control this potential second pandemic wave, national and local authorities implemented a series of mitigation measures in certain metropolitan areas starting mid-October. A countrywide lockdown followed on 30 October. Here we assess the impact and timeliness of these measures, mainly curfews, by conducting a descriptive temporal analysis of indicators reflecting COVID-19 spread and severity in the 22 French metropolitan areas. Altogether these areas host 28.5% of the French population, and 18 of them had been targeted by curfews prior to the national lockdown in autumn.

## Increasing series of mitigation measures to control the second wave

On 17 October, a curfew was implemented from 9 p.m. to 6 a.m. in the nine most affected metropolitan areas. Reinforced measures such as limiting public and private social gatherings, closing bars and/or restaurants, or prohibiting alcohol sales in public areas had already been put in place in these nine metropolitan areas since 23–25 September. One week later, on 24 October, the curfew was extended to nine additional metropolitan areas where viral transmission was also critically increasing. Finally, on 30 October, following a concerning increase in COVID-19-related hospital and intensive care unit (ICU) admissions and deaths, a nationwide lockdown was implemented. This was switched to a national curfew on 15 December 2020, which is still ongoing as at 17 December.

## Timing of mitigation measures and evolution of the COVID-19 epidemic in metropolitan areas

All laboratory-confirmed cases (patients with a newly positive SARS-CoV-2 real-time (RT)-PCR on a nasopharyngeal swab, thereafter referred to as ‘confirmed cases of COVID-19’) and hospitalised cases were analysed according to the date of sample collection and hospital admission, using routine COVID-19 surveillance tools. Seven-day rolling incidence of confirmed cases’ and hospital admissions’ rates, as well as test (RT-PCR) positivity rates, were calculated daily for each metropolitan area. Temporal evolution of these parameters was described among three groups of metropolitan areas, constituted according to the measures implemented and their timing (Table 1). Timing of mitigation measures were considered as the date of their implementation but, based on the natural history of SARS-CoV-2 infection and data on sampling delay, their effects are expected to be observable at least 1 week later [2,3].

**Table 1:** Description of the three groups of metropolitan areas concerned by mitigation measures before 15 December, France, autumn 2020 (n = 22 metropolitan areas)

Characteristics	Group 1^a^ Under curfew since 17 October	Group 2 Under curfew since 24 October	Group 3 Without curfew
Number of metropolitan areas	9	9	4
Population	14,014,489	3,278,393	1,853,088
List of metropolitan areas	Grenoble-Alpes-Métropole Métropole Européenne de Lille Métropole de Lyon Métropole d'Aix-Marseille-Provence Montpellier Méditerranée Métropole Métropole du Grand Paris Métropole Rouen Normandie Saint-Etienne Métropole Toulouse Métropole	Clermont Auvergne Métropole Dijon Métropole Métropole du Grand Nancy Métropole Nice Côte d'Azur Orléans Métropole Rennes Métropole Eurométropole de Strasbourg Métropole Toulon-Provence-Méditerranée Tours Métropole Val de Loire	Bordeaux Métropole Brest Métropole Metz Métropole Nantes Métropole

^a^ These areas were already concerned, since 23–25 September, by reinforced measures such as limitation of public and private social gatherings, closure of bars and/or restaurants, or prohibition of alcohol sales in the public domain.

**Figure 1:**
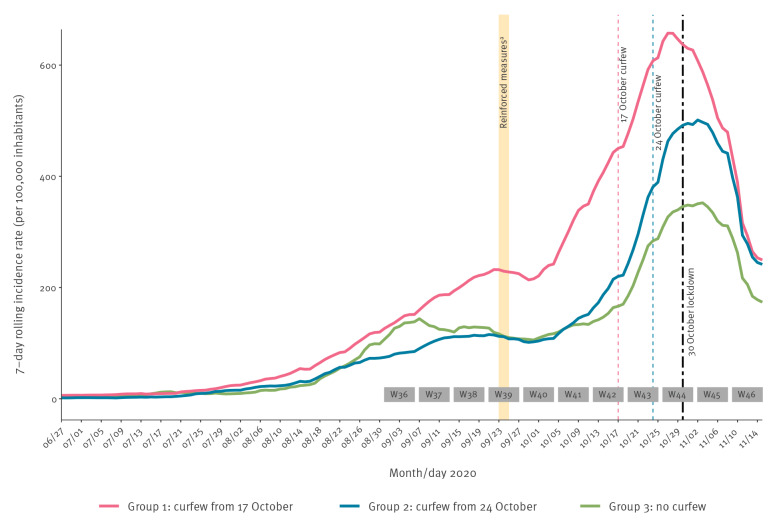
Evolution of 7-day rolling incidence rate of confirmed cases of COVID-19 by group of metropolitan areas, France, 27 June–15 November 2020 (n = 3 groups of metropolitan areas)

COVID-19: coronavirus disease; W: week.

^a^ These measures only apply to Group 1, and to one metropolitan area of Group 3 (Bordeaux).

**Figure 2:**
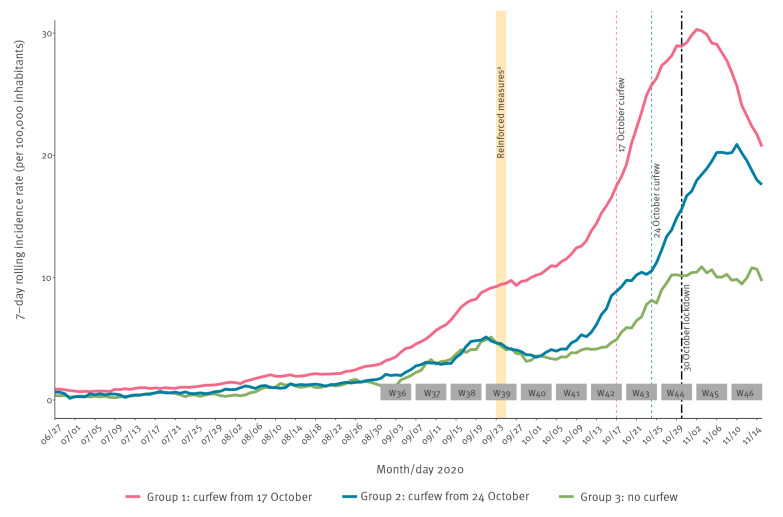
Evolution of 7-day rolling rate of hospital admissions for COVID-19 by group of metropolitan areas, France, 27 June–15 November 2020 (n = 3 groups of metropolitan areas)

COVID-19: coronavirus disease; W: week.

^a^ These measures only apply to Group 1, and to one metropolitan area of Group 3 (Bordeaux).

As shown in Figure 1 and Figure 2, incidence and hospitalisation rates dramatically increased from the beginning of October, especially in areas characterised by the highest incidence (Group 1), justifying the implementation of measures at an earlier time for such areas. Regarding incidence rate of confirmed cases of COVID-19 (Figure 1), in Group 1 (curfew implemented on 17 October), the peak was reached on 27 October. In Groups 2 (curfew implemented on 24 October) and 3 (no curfew), the increase slowed down as early as the end of October, and the peak was reached 1 week later, on 2 and 3 November, respectively. In the three groups, the change in incidence slope was followed by a rapid and marked decrease.

The 30 October lockdown, represented by a black vertical dotted line, is a nationwide lockdown.

The 30 October lockdown, represented by a black vertical dotted line, is a nationwide lockdown.

In Groups 1 and 2 (Figure 2), hospital admissions rates followed a pattern similar to the incidence rates with a lag of a few days and peaks on 2 and 10 November respectively, followed by a rapid decrease. In Group 3, hospital admissions plateaued from the end of October to mid-November.

In Table 2, we quantified changes in epidemiological rates across the three groups using the weekly percentage variations of incidence, test positivity and hospital admissions rates, between weeks 40 (28 September–4 October) and 46 (9–15 November). In the three groups, a slowdown in the increase of incidence rate was observed in week 44, when the impact of the first curfew could theoretically be expected, followed by a decrease in week 45, particularly marked in Group 1 (− 24%). In week 46, i.e. 10 days after implementation of lockdown, the decrease was considerable in the three groups. Similar patterns with time were observed for positivity rate, with a marked reduction for the three groups in week 46 (− 15.7 to − 20.6%). Regarding hospital admissions, a slowdown was observed from week 44 in Group 1, and from week 45 in Groups 2 and 3; followed by a clear decrease in Groups 1 and 2, and a stabilisation in Group 3.

**Table 2:** Weekly variations of confirmed cases of COVID-19, positivity and hospital admissions rates, by metropolitan areas groups, France, 28 September–15 November 2020

Characteristic			Week 40 28 Sep–4 Oct	Week 41 5 Oct–11 Oct	Week 42^a^ 12 Oct–18 Oct	Week 43^b^ 19 Oct–25 Oct	Week 44^c^ 26 Oct–1 Nov	Week 45 2 Nov–8 Nov	Week 46 9 Nov–15 Nov
Confirmed cases per 100,000 inhabitants	Group 1^a,c^	Incidence rate	242.2	350.2	453.6	613.2	627.0	479.4	249.6
		W vs W − 1 (%)	NA	+ 44.6	+ 29.5	+ 35.2	+ 2.2	− 23.5	− 47.9
	Group 2^b,c^	Incidence rate	108.5	151.8	222.3	389.3	493.1	441.5	241.7
		W vs W − 1 (%)	NA	+ 39.9	+ 46.4	+ 75.1	+ 26.7	− 10.5	− 45.3
	Group 3^c^	Incidence rate	117.1	133.6	170.1	288.0	346.9	310.9	173.7
		W vs W − 1 (%)	NA	+ 14.2	+ 27,3	+ 69.3	+ 20.5	− 10.4	− 44.1
Test (RT− PCR) positivity	Group 1^a,c^	Rate (%)	13.6	16.8	17.9	22.4	23.7	22.2	17.6
		W vs W − 1 (%)	NA	+ 23.7	+ 6,4	+ 24,9	+ 5,9	− 6.3	− 20.6
	Group 2^b,c^	Rate (%)	7.5	9.6	11.5	15.9	18.9	18.9	15.0
		W vs W − 1 (%)	NA	+ 28.3	+ 20.4	+ 38.2	+ 18.5	− 0.2	− 20.3
	Group 3 ^c^	Rate (%)	9.2	9.7	10.2	14.7	16.3	15.3	12.9
		W vs W − 1 (%)	NA	+ 4.8	+ 5.3	+ 44.3	+ 10.8	− 5.8	− 15.7
Hospital admissions per 100,000 inhabitants	Group 1^a,c^	Incidence rate	10.9	12.9	18.1	26.1	29.8	27.6	20.6
		W vs W − 1 (%)	NA	+ 18.6	+ 40.2	+ 44.1	+ 13.9	− 7.4	− 25.4
	Group 2^b,c^	Incidence rate	4.1	5.1	9.2	11.2	16.4	20.0	17.5
		W vs W − 1 (%)	NA	+ 25.9	+ 79.4	+ 21.3	+ 46.8	+ 22.2	− 12.7
	Group 3^c^	Incidence rate	3.3	4.1	5.4	7.7	10.3	10.0	9.5
		W vs W − 1 (%)	NA	+ 23.8	+ 33.3	+ 42.3	+ 32.4	− 2.0	− 5.2

COVID-19: coronavirus disease; NA: non-applicable (as outside the period of observation); W: week.

^a^ Group 1: curfew from 17 October.

^b^ Group 2: curfew from 24 October.

^c^ Group 1, 2, and 3: lockdown from 30 October.

## Ethical statement

An ethical approval for this study was not necessary, because data were not identifiable back to the patients from whom they came from.

## Discussion

### Can we believe that the measures were effective?

The change in incidence and hospital admissions slopes, observed 7 to 10 days after implementation of mitigation measures coupled with an intense communication on the severity of the epidemic, is consistent with a possible positive impact of these actions. Changes occurred first in the metropolitan areas where reinforced measures and curfew were initially implemented (17 October). About 10 days after the national lockdown was in place, a similar marked decrease in incidence rate was obvious across all groups of metropolitan areas, i.e. whatever the measures prior implemented. These temporal coincidences suggest a positive impact of curfew and lockdown, which is consistent with international experiences [2-8].

Other factors may have also contributed to the observed positive evolution, notably school holidays from 17 October to 1 November, whose start coincided with the announcement of the first curfew, likely led to a decrease in social interactions, as previously described for respiratory infections such as influenza or respiratory syncytial virus (RSV) [9]. Nevertheless, the favourable developments observed, despite the end of the school holidays and before possible impact of the nationwide lockdown, suggest that curfews, intense communication about the severity of the pandemic and other local mitigation measures (limiting public and private social gatherings, closing bars and/or restaurants, prohibiting alcohol sales) might have played a considerable role. No noteworthy changes in screening strategy and/or access to testing since the measures were implemented occurred that could explain the observations in this study. Furthermore, the decline of test positivity rate and incidence of hospital admissions speaks in favour of a real decrease in viral transmission.

### Were the effects restricted to targeted areas?

In metropolitan areas not concerned by the first curfew (Groups 2 and 3), an improvement of the epidemiological situation was observed during the end of October. As measures for these areas only occurred from 24 October, their effect would not have been foreseen to occur until at least 1 week later. The hypothesis of an impact of the first curfew (and earliest reinforced measures at the end of September) in these areas not directly targeted by the measure, through a ‘resonance effect’, can be raised. Thus, the first curfew could have had an impact in more areas than the ones targeted, resulting in behavioural changes and then a decrease in viral transmission. The intense communication on the severity of the epidemic in the whole country at the time of curfews might have also led to behavioural changes nationwide.

## Conclusion

This early descriptive analysis is suggestive of a positive impact of mitigation measures implemented to face the emergence of a second wave of the COVID-19 pandemic in France. Indeed, a considerable decrease in incidence and hospital admissions was observed 7 to 10 days after the measures were put in place, occurring earlier in metropolitan areas where these had first been undertaken. Continued analysis of the epidemiological evolution within the next weeks will help to clarify the specific role of these measures and guide future public health decisions. An analytic approach including time-series and geographical modelling will be of interest to take into account other factors (holidays, screening and contact-tracing activities, adherence to measures, meteorological factors, etc.) that may have influenced the dynamic of the epidemic. Social sciences could also be helpful to understand public attitudes and perceptions leading to behavioural changes across all groups, in order to assess the possibility of a ’resonance effect’.
